# Impacts of asymmetric hip rotation angle on gait biomechanics in patients with knee osteoarthritis

**DOI:** 10.1186/s43019-024-00226-5

**Published:** 2024-07-14

**Authors:** Ji-yeon Yoon, Sang Won Moon

**Affiliations:** 1 Department of Physical Therapy, Inje University Haeundae Paik Hospital, Busan, Korea; 2 Department of Orthopedic Surgery, Inje University Haeundae-Paik Hospital, 875 Haeun-Daero, Haeundae-Gu, Busan, 48108 Korea

**Keywords:** Knee osteoarthritis, Knee adduction moment, Hip rotation, Pelvic lateral tilt, Gait

## Abstract

**Background:**

Knee Osteoarthritis (OA) is a highly prevalent age-related disease. The altered kinematic pattern of the knee joint as well as the adjacent joints affects to progression of knee OA. However, there is a lack of research on how asymmetry of the hip rotation angle affects the gait pattern in knee OA patients.

**Research question:**

What are the impacts of asymmetric hip rotation range on gait biomechanical characteristics and do the gait patterns differ between patients with knee OA and healthy elderly people?

**Methods:**

Twenty-nine female patients with knee OA and 15 healthy female elders as control group were enrolled in this study. The spatiotemporal parameters, kinematic and kinetic data during walking were measured using a three-dimensional motion capture system. The differences between knee OA and control group were analyzed using an independent *t*-test.

**Results:**

The knee OA group exhibited a significant reduction in hip internal rotation range and internal/external rotation ratio on more affected side (p < 0.05). Significant differences were found in spatiotemporal parameters except to the step width. Significant reductions were also found in kinematic parameters (pelvic lateral tilt range, sagittal angle ranges in hip, knee and ankle, knee adduction mean angle). There were also significant differences in vertical ground reaction force and knee adduction moment (p < 0.05).

**Conclusions:**

Knee OA patients have asymmetric hip rotation ranges. Especially limited hip internal rotation could lead to the reduction of pelvic lateral tilt, which may cause greater knee joint loading. Therefore, it is necessary to pay attention to recovery of hip rotation after knee surgery.

## Introduction

Knee osteoarthritis (OA) is the most common progressive disease among elderly people that causes difficulty in physical activities such as walking, sit to standing and descending stairs due to pain, joint stiffness and instability, and muscle weakness, all of which reduce quality of life [1]. Knee adduction moment (KAM) which provides an indication of the actual loads at the knee joint is recognized as a major clinical marker of knee OA progression [2, 3]. Many previous studies reported that poor alignments of the knee joint by tibial torsion or tibial varus deformity make people more liable to developing the knee OA and knee load increase [4, 5]. Therefore, higher dynamic knee loading is related to the progression of knee OA [6]. The movement of the human body is connected, so when a problem occurs in one joint, it can affect the other [7].

The foot posture and kinematics of the ankle joint, which is the distal joint of the knee affect the movement of the lower extremities during walking. Many studies have shown that foot progression angle is closely related to KAM [8–10]. The increased foot progression angle is known as the gait pattern to reduce increased KAM or to maintain mediolateral stability [11, 12]. The hip is the proximal joint of the knee. It affects the knee joint function and loading due to sharing a common segment with the knee [13, 14]. The angle of internal rotation and external rotation of the hip joint is the same at 45 degrees and is connected to each other, so if the internal rotation angle becomes small, the external rotation angle will increase [14]. Kim et al [15] reported that asymmetric hip rotation ranges increased the peak of the KAM.

Altered hip kinematics are associated with musculoskeletal problems, especially affecting adjacent joints such as knee pain and lower back pain. Until now, studies related to hip rotation asymmetry have been limited to young adults and athletes [15–18]. Although there are many patients who have difficulty walking with knee OA as well as lower back pain as they get older, studies related to hip rotation range in the elderly are insufficient. Therefore, the aim of this study is to investigate the range of hip internal rotation (IR) and external rotation (ER) and to find out the effect on gait patterns in knee OA patients. Our hypothesis was that hip internal rotation and lower limb joint angle in sagittal plane would decrease, and KAM, knee adduction angle and foot progression angle would increase in patients with knee OA.

## Methods

### Subjects

The power analysis was performed to determine the sample size for this study by using G-Power software (ver. 3.1.9.7; Franz Faul, University of Kiel, Germany). The effect size determined from a pilot study with 7 people in each group was 2.6. The calculated sample size was 12 (knee OA group, *n =* 6; control group, *n =* 6) with a significance level of 0.05, power of 0.95, and effect size of 2.6. This study reviewed 29 female patients with moderate to severe medial knee OA who were waiting to undergo total knee arthroplasty (TKA) surgery. We only enrolled female patients as subjects to remove the bias associated with the biomechanical gender differences. The following inclusion criteria were: (1) women aged 60 years or older; and (2) diagnosed with knee OA in the medial compartment with classified as Kellgren-Lawrence grade 3 (moderate) or 4 (severe). The exclusion criteria were: (1) concurrent back pain or any neurological disorders that could affect independent gait; (2) more osteophytes in the lateral compartment; and (3) rheumatoid arthritis. Fifteen age-matched healthy women were recruited as control group. The inclusion criteria were: (1) no clinical diagnosis of knee OA; (2) no neurological disorders or musculoskeletal disease; and (3) no history of lower limb surgery. All subjects signed the informed consent form, which was approved by the Haeundae-paik hospital Ethics Committee for Human Investigations.

### Measurement protocol

We used a standard 12-inch plastic round universal goniometer to measure passive hip rotation range of motion. When measuring hip rotation, subjects were placed in the supine position on an examining table and they wore short pants. Leg alignment was visualized with two reference points (on the anterior tibial tuberosity and at the intersection of the bimalleolar line and the anterior crest of the tibia). The hip being measured was flexed to 90° and the contralateral hip was placed in neutral. The knee was flexed to 90° and the examiner moved ankle to produce hip rotation. The pelvic was stabilized with a strap firmly tightened over the sacrum to prevent the pelvic movement [17]. Through the above process, we acquired the hip IR and ER range and calculated additionally IR/ER ratio for easy comparison of the changed angles between IR and ER.

A VICON motion capture system (VICON Motion Systems Ltd, Oxford, UK) was used to collect the gait parameters including kinetic and kinematic data. Prior to the gait test, 16 retro-reflective markers (diameter of 14 mm) were placed on the patient. The locations of markers were based on the Plug-in-Gait model marker set for lower limb biomechanics (bilateral anterior and posterior superior iliac spines, lateral thigh, femoral epicondyle, tibia malleolus, second metatarsal head, and posterior calcaneus). All markers were fixed by the same examiner. After a static standing posture was captured, the patient was asked to walk along an 8-m walkway with two ground-embedded force plates (AMTI, Advanced Mechanical Technology Inc., Watertown, MA, USA) in the middle.

The marker data were synchronized with the kinematic data via Nexus software (VICON, version 1.7) and filtered at 6 Hz using a zero-lag, bidirectional second-order Butterworth filter. The kinetic data including knee adduction moment (KAM) and ground reaction force (GRF) were recorded using two force plates with a sampling rate of 1,000 Hz and processed using a low-pass filter with a cut-off frequency of 50 Hz. Spatiotemporal parameters, kinematic and kinetic data were calculated for each patient according to her anthropometric characteristic. Each subject walked at preferred speed until at least four gait cycles had been completed and were repeated more than three trials separately. Analyses were performed only for the leg scheduled for TKA surgery and if both legs were planned for surgery, more affected leg was selected for analysis. Only data on which each foot was precisely stepped on the force plate were selected, and two gait trials were averaged and analyzed. The pelvic lateral tilt and rotation, sagittal plane hip and knee range was calculated as maximum minus minimum value throughout gait cycle. Sagittal plane ankle range was calculated as maximum minus minimum value during mid- to terminal stance phase. Knee adduction and foot progression angle was calculated as mean value during stance phase. Peak external KAM and ground reaction force (GRF) was identified during stance phase. Acquired gait data were analyzed by using Polygon software (VICON, version 3.1) and the gait data were time normalized to the gait cycle. The KAM was normalized for body weight and height (%BW·Ht), and GRF was normalized to the body weight (%BW).

### Statistical analysis

SPSS software (version 22.0, Chicago, IL, USA) was used for statistical analyses. Data were normally distributed, thus the general participant characteristics and gait parameters from the two groups were compared using independent *t*-tests. The level of statistical significance was considered value of p less than 0.05.

## Results

There were significant differences in the mechanical axis and body mass index of demographic characteristics (Table 1). Significant reductions were in hip internal rotation range (*p* = 0.001) and IR/ER ratio (*p* = 0.003) between more affected side in knee OA group and right side in control group (Table 2).

**Table 1:** General information between knee OA and control group

	Knee OA (*n =* 29)	Controls (*n* = 15)	Effect size	*P*
	Mean (SD)	Mean (SD)		
Age (years)	71.7 (5.5)	70.5 (6.0)	0.207	0.512
Height (m)	1.54 (0.06)	1.57 (0.06)	− 0.451	0.157
Weight (kg)	65.1 (8.0)	59.9 (9.3)	0.605	0.057
BMI (kg/m^2^)	27.6 (3.6)	24.4 (3.8)	0.848	**0.010**
Mechanical axis (°)	9.2 (4.4)	0.5 (2.4)	2.455	**0.000**
K-L grade 3/4 (n)	16/13	–	–	–

Statistically significant parts of the p values are highlighted in bold

BMI, body mass index; K-L grade, Kellgren-Lawrence grade; SD, standard deviation

**Table 2:** Hip rotation angle and ratio between Knee OA and control group

Variables	Side	Knee OA (*n* = 29)	Controls (*n* = 15)	Effect size	*P*
		Mean (SD)	Mean (SD)		
Hip IR (degrees)	More affected (Right) * side	26.34 (7.72)	34.13 (5.84)	− 1.138	**0.001**
	Less affected (Left) side	30.00 (7.63)	33.93 (10.11)	− 0.439	0.155
Hip ER (degrees)	More affected (Right) side	46.21 (8.93)	45.80 (7.19)	0.051	0.880
	Less affected (Left) side	45.10 (9.12)	49.00 (8.49)	− 0.443	0.177
IR/ER ratio	More affected (Right) side	0.59 (0.18)	0.76 (0.17)	− 1.004	**0.003**
	Less affected (Left) side	0.69 (0.23)	0.71 (0.23)	− 0.092	0.775

Statistically significant parts of the p values are highlighted in bold

ER, external rotation; IR, internal rotation; SD, standard deviation

^*^(Right or Left) indicates the leg side of the control group

In spatiotemporal parameters, we found that the walking speed, cadence and step length were decreased in knee OA group than in control group (p<0.05). There was no significant difference in step width between two groups (Table 3).

**Table 3:** Spatiotemporal parameter, kinematic, and kinetic data between Knee OA and control group

	Knee OA (*n* = 29)	Controls (n = 15)	Effect size	*P*
	Mean (SD)	Mean (SD)		
**Spatiotemporal parameters**				
Walking speed (m/s)	0.59 (0.22)	1.02 (0.13)	− 2.419	**0.000**
Step time (s)	0.74 (0.27)	0.54 (0.04)	1.048	**0.006**
Step length/height ratio	0.26 (0.08)	0.35 (0.03)	− 1.587	**0.000**
Step width (m)	0.16 (0.05)	0.15 (0.03)	0.297	0.380
Cadence (steps/min)	87.55 (17.16)	111.55 (8.56)	− 1.771	**0.000**
**Kinematics**				
Pelvic lateral tilt range throughout gait cycle (°)	5.83 (2.99)	7.95 (2.37)	− 0.786	**0.022**
Pelvic rotation range throughout gait cycle (°)	8.56 (3.85)	8.01 (3.03)	0.16	0.631
Hip sagittal range throughout gait cycle (°)	36.64 (9.72)	44.17 (8.69)	− 0.817	**0.016**
Knee sagittal range throughout gait cycle (°)	34.86 (8.91)	53.44 (3.59)	− 2.735	**0.000**
Ankle sagittal range during mid- to terminal stance phase (°)	25.81 (6.76)	32.00 (6.06)	− 0.964	**0.005**
Knee adduction mean angle during stance phase (°)	5.87 (5.70)	− 4.24 (4.00)	2.054	**0.000**
Foot progression mean angle during stance phase (°)	7.16 (7.21)	8.42 (4.94)	− 0.204	0.549
**Kinetics**				
Peak vertical ground reaction force (%BW)	100.34 (3.30)	109.60 (5.60)	− 2.013	**0.000**
Peak knee adduction moment (% Ht·BW)	4.99 (1.84)	3.71 (1.94)	0.645	**0.037**

Statistically significant parts of the p values are highlighted in bold

BW, body weight; Ht, height; SD, standard deviation

In kinematic and kinetic data, we found that pelvic lateral tilting range throughout gait cycle was significantly smaller in knee OA group than in control group (*p* = 0.022). Mean value of knee adduction angle during stance phase was significantly greater in knee OA group than in control group (*p* = 0.000). There were no significant differences in pelvic rotation range and foot progression angle between two groups. Peak vertical ground reaction force was significantly lower (*p* = 0.000) and KAM was significantly higher (*p* = 0.037) in knee OA group than in control group (Table 3).

## Discussion

The most important finding of this study is that knee OA patients have asymmetric hip rotation range and IR/ER ratio. This was mostly consistent with our hypothesis, but the foot progression angle was not.

The hip internal rotation angle and IR/ER ratio on more affected side of knee OA group was significantly smaller than it on the right side of control group. This is similar to the previous results. Kim et al [15] showed that people who responded to the specialized mobility footwear had properties more related to the knee OA progression. The responders showed lower hip IR range and IR/ER muscle strength ratio. Cibulka et al [17] reported that the less hip IR range compared to ER often show the weakness of the hip internal rotator muscles. The larger difference between hip IR and ER range is associated with a more mal-alignment of the lower limb such as genu varum and valgus, pes planus [16, 19].

One of interesting findings of this study was that patients with knee OA with reduced hip IR showed significantly decreased pelvic lateral tilting range compared to control group. The hip internal rotation and pelvic 4° lateral tilting during loading response phase plays roles as absorbing the impact of the joint and helps the contralateral pelvic advancing. Hayot et al [20] emphasized that the pelvic lateral tilting has an important influence as strategies to minimize energy consumption during walking. Therefore, limiting the internal rotation of the hip joint leads to the limitation of the pelvic drop, which increases knee joint loading and prevents contralateral leg advancing [21]. In addition, if the external rotation movement is repeated due to the limitation of internal rotation of the hip joint, the stress on the ligaments around the hip and knee may increase. This repeated hip external rotation seems to have contributed to increasing pelvic backward rotation. The hip extensors and abductors play a primary action to make step length through late terminal swing to midstance phase [21]. In our study, there was no significant difference in pelvic rotation between groups. We consider that patients with knee OA may increase the pelvic rotation during gait to compensate the reduced sagittal hip and knee joint angles [22].

As the previous studies on gait analysis in patients with knee OA [23–25], walking speed, step length and cadence decreased and kinematic ranges of lower limb joints decreased, and KAM and knee adduction angle increased compared to the controls. Abnormal gait patterns are related to the knee pain and muscle weakness, and greater knee varus alignment led to the less knee flexion and greater KAM [12, 26, 27]. Since GRFs shows a strong correlation with faster walking speed [28], it could be expected that vertical GRF in knee OA patients would show a significant decrease. The KAM is calculated by GRF and its lever arm which the perpendicular distance between the vector line and the knee joint center [29]. Therefore, a significant increase in moment arm due to knee varus alignment would have contributed to knee OA progression, which showed greater KAM despite a significantly decreased GRF. In this study, only step width was no significant difference among spatiotemporal parameters. Elderly adults usually walk with wider step width compared to the youngers to ensure the dynamic stability [30]. According to a recent study [31], step width among people with knee OA affects trunk and gait patterns, and in this study, all patients with wide or narrow step width were included, so it seems that there is no difference from the control group.

Contrary to the hypothesis that foot progression angle would increase in knee OA, we found that there was no significant difference between the two groups. Many previous studies have reported that out toeing gait compensate to reducing KAM [12, 32, 33], but a study has reported that KAM decreases during in-toeing gait [34]. Previous studies have shown that FPA in knee OA patients is still controversial. In our study, there was wide distribution in FPA including 4 patients with toe-in angle. Also, the external foot rotation appears as one of the compensation strategies for front plane stability walking. In this study, there was no significant difference in step width, so it is thought that it did not affect FPA. Yoon & Shin [31] reported that among female OA patients, those with wider step width showed greater foot progression angles. In addition, a recent study reported that external tibial torsion was related to external foot progression [35], but it is difficult to compare because external tibial torsion as one of our study limitations is not measured in this study.

There were several limitations in this study. First, there was a difference in BMI between the two groups. BMI is one of the important risk factors for knee OA. Therefore, it may affect the results of this study. Second, we did not include patients with mild knee OA because our subjects were composed of female patients with knee OA requiring TKA surgery. Therefore, there is a limit to the application to all patients with knee OA. Third, only the affected legs were compared, and the compensatory movement of contralateral side was not included in this study. Forth, we did not consider the patients’ hip anteversion that could affect hip rotation angle. Last, only the angles of hip IR and ER were compared without measuring the internal and external rotation muscle strength of the hip joint. Hip rotation cannot be separated from the role of the muscles involved. Therefore, additional study is needed to interpret hip rotation in patients with knee OA based on the dynamic role of muscles.

## Conclusion

This study compared the hip rotation angles between patients with knee OA and controls, and investigated how asymmetric hip rotation angle affects the gait pattern in patients with knee OA. The patients with knee OA had asymmetric hip rotation ranges. Especially limited hip internal rotation could lead to the reduction of pelvic lateral tilt, which may cause greater knee joint loading during walking. Therefore, it is necessary to pay attention to recovery of hip rotation after knee surgery.

## Data Availability

Not applicable.
